# Geographic variation in inpatient medical expenditure among older adults aged 75 years and above in Japan: a three-level multilevel analysis of nationwide data

**DOI:** 10.3389/fpubh.2024.1306013

**Published:** 2024-02-28

**Authors:** Yuki Shirakura, Yugo Shobugawa, Reiko Saito

**Affiliations:** ^1^Division of International Health (Public Health), Graduate School of Medical and Dental Sciences, Niigata University, Niigata, Japan; ^2^Department of Active Ageing, Graduate School of Medical and Dental Sciences, Niigata University, Niigata, Japan

**Keywords:** medical expenditure, geographic variation, regional disparities, older adults, population ageing, multi-level analysis, Japan

## Abstract

**Introduction:**

In Japan, a country at the forefront of population ageing, significant geographic variation has been observed in inpatient medical expenditures for older adults aged 75 and above (IMEP75), both at the small- and large-area levels. However, our understanding of how different levels of administrative (geographic) units contribute to the overall geographic disparities remains incomplete. Thus, this study aimed to assess the degree to which geographic variation in IMEP75 can be attributed to municipality-, secondary medical area (SMA)-, and prefecture-level characteristics, and identify key factors associated with IMEP75.

**Methods:**

Using nationwide aggregate health insurance claims data of municipalities for the period of April 2018 to March 2019, we conducted a multilevel linear regression analysis with three levels: municipalities, SMA, and prefectures. The contribution of municipality-, SMA-, and prefecture-level correlates to the overall geographic variation in IMEP75 was evaluated using the proportional change in variance across six constructed models. The effects of individual factors on IMEP75 in the multilevel models were assessed by estimating beta coefficients with their 95% confidence intervals.

**Results:**

We analysed data of 1,888 municipalities, 344 SMAs, and 47 prefectures. The availability of healthcare resources at the SMA-level and broader regions to which prefectures belonged together explained 57.3% of the overall geographic variance in IMEP75, whereas the effects of factors influencing healthcare demands at the municipality-level were relatively minor, contributing an additional explanatory power of 2.5%. Factors related to long-term and end-of-life care needs and provision such as the proportion of older adults certified as needing long-term care, long-term care benefit expenditure per recipient, and the availability of hospital beds for psychiatric and chronic care and end-of-life care support at home were associated with IMEP75.

**Conclusion:**

To ameliorate the geographic variation in IMEP75 in Japan, the reallocation of healthcare resources across SMAs should be considered, and drivers of broader regional disparities need to be further explored. Moreover, healthcare systems for older adults must integrate an infrastructure of efficient long-term care and end-of-life care delivery outside hospitals to alleviate the burden on inpatient care.

## Introduction

Over the past three decades, Japan’s national medical expenditures have nearly doubled, reaching around 43 trillion yen (304 billion USD) in 2020 (1). This translates to an increase from 4.6 to 8.0% in the country’s gross domestic product since 1990. The ageing population is a cardinal contributor to this rapid rise, as *per capita* healthcare costs increase with advancing age and healthcare resources are utilised disproportionately by older adults (2, 3). As of 2020, the population aged 65 and above (28.6% of the total population) accounted for 61.5% of the national medical expenditure in Japan (1, 4).

Japan achieved universal health insurance coverage in 1961, following the establishment of employer-based insurance for the employed and the National Health Insurance for the self-employed, unemployed, and retired (5). In anticipation of an ageing population, Japan underwent a health insurance reform in 2008, establishing the Medical Care System for Older Adults Aged 75 and Over (MCS75)—a new national health insurance scheme, specifically designed for adults aged 75 and above (6). Upon reaching the age of 75 years, all individuals are withdrawn from their current public health insurance coverage and uniformly enrolled in the MCS75, regardless of their employment or health status. An exception to this enrolment is welfare recipients, whose healthcare expenses are covered through public assistance. Additionally, those aged 65 to 74 years with certain disabilities are eligible for coverage by the MCS75. In 2018, 17.4 million people were insured by the MCS75, of whom 98.2% (17.1 million) were people aged 75 and above. This represents 99.1% of adults aged 75 and above in Japan, based on the resident register (7).

Geographic variation in medical expenditure has been well recognised in Japan (8, 9), despite the country’s universal health insurance system, uniform fee schedules, and national-level policies guiding the design of the healthcare infrastructure (6). Such geographic variation is strongly evident in the care of older adults aged 75 years and above, and is largely attributable to heterogeneity in hospitalisation expenses (9). Inconsistencies in the number of inpatient beds, notably for psychiatric and chronic care, have been criticised as a key driver of geographic disparities in inpatient care costs for older adults (10). Excess bed availability in some areas increases the likelihood of them functioning as long-term care homes, leading to extended hospitalisation beyond medical needs (11). While the phenomenon of supplier-induced demand in healthcare utilisation has been well documented elsewhere (12–15), European and US studies report health, sociodemographic, and cultural factors as important determinants of geographic variations in healthcare costs (16–18). However, there remains a paucity of evidence to determine the degree to which the level of healthcare demand, as influenced by population characteristics, contributes to geographic differences in Japan’s medical expenditure.

Geographic disparities in medical expenditure have been observed across various administrative divisions in Japan, including municipalities, secondary medical areas (SMAs), prefectures, and broader regional levels, with a tendency for healthcare spending to be higher in the western and northern regions, and lower in eastern Japan (9). However, previous studies have primarily focused on large-area analyses (8, 10, 19), or a specific part of Japan (20), and our understanding is limited regarding the contribution of each administrative level to the overall geographic variation and the factors influencing them.

To address the research gaps in the geographic disparities in inpatient medical expenditure, particularly for older adults aged 75 years and above covered by the MCS75, this study aimed to (1) quantify the magnitude of the geographic variation at each level of healthcare administration, namely the municipalities, SMAs, and prefectures; (2) determine the extent to which geographic variation can be attributed to the availability of healthcare resources and factors influencing healthcare demand; and (3) identify key factors influencing inpatient medical expenditure. To this end, we employed a multilevel modelling approach that incorporated the hierarchical structure of healthcare administration.

Japan is currently implementing a healthcare system reform, known as the “Regional Medical Care Vision,” in an effort to address the needs of the ageing population, particularly the baby boomer generation, as they reach the age of 75 or above by 2025 (21). Under this government policy, prefectures are tasked to estimate the number of hospital beds required for acute, subacute, and chronic care in each SMA by 2025, using population projections and nationwide standardised formulae (22). Any bed excess or shortage is encouraged to be rectified through restructuring of hospital bed capacity and hospital reorganisation within the SMA. While this initiative may contribute to addressing regional disparities in hospital expenses through the optimisation of healthcare delivery within SMAs, it carries the risk of further depriving areas that already have limited healthcare resources and jeopardising equity in healthcare access by hasty hospital reorganisation. In this context, geographic variation in medical expenditure is an issue of inefficiency which, if left unattended, could give rise to inappropriate health financing, ineffective resource allocation, and avoidable waste (16). On the other hand, it is also an issue of inequality, where uneven healthcare access and quality could lead to regional disparity in population health (23–25). Thus, a sound understanding of the determinants of geographic variation in medical expenses is essential to rationally assess the impacts of the Regional Medical Care Vision on healthcare delivery and guide evidence-informed health policymaking.

## Methods

### Study design and setting

This nationwide cross-sectional ecological study used municipality-level medical expenditure data from the Medical Care System for Older Adults Aged 75 and Over (MCS75) in Japan, which is publicly available from an online government source (9). We focused our analysis on this population, as the MCS75 data contains insurance claims data for virtually all individuals aged 75 or above in Japan, ensuring the generalizability of the study findings to the entire country. Individuals aged 74 and below are covered by various health insurance schemes, and much of this data is not publicly available.

Administratively, Japan has two tiers of local governance: 47 prefectures and 1,741 municipalities, including cities, towns, and villages. In healthcare administration, the Ministry of Health, Labor, and Welfare (MHLW) devises healthcare policies and operates through seven Regional Health and Welfare Bureaus (RHWBs) that oversee service providers within their respective regions (RHWB regions), namely, Hokkaido, Tohoku, Kanto-Shinetsu, Tokai-Hokuriku, Kinki, Chugoku-Shikoku, and Kyushu. Each RHWB region encompasses one or more prefectures. Background information on the RHWB regions, including a summary of regional populations and gross regional products is provided in Supplementary Table 1. At the local level, prefectures are tasked with planning and developing healthcare delivery systems. To this end, each prefecture establishes SMAs, which are typically composed of one to several municipalities and serve as the fundamental units for designing and coordinating the provision of integrated inpatient care services for residents, including acute, subacute, and chronic care. As of 2017, there were 344 SMAs in the country.

Initially, we obtained data for all 1,741 municipalities in Japan. The 20 major cities designated under special government ordinances were subdivided into smaller administrative units called wards. Since ward-level data for medical expenditures were publicly available, the 175 wards were treated as separate municipalities instead of the 20 cities. In cases where ward-level data were unavailable for certain explanatory variables, the corresponding city-level values were used as substitutes. Municipalities with incomplete data were excluded from final analyses.

### Data sources and variables

We accessed the MHLW website to obtain data on municipality-level inpatient medical expenditures for older adults aged 75 and above (IMEP75) in Japanese Yen (JPY)—the outcome variable of this study. These datasets are compiled annually by the MHLW from the National Database of Health Insurance Claims and Specific Health Checkups of Japan, in which the claims data of the MCS75 are accumulated (9). IMEP75 includes the costs of treatment for all health conditions, as well as meal and living expenses during hospitalisation which are covered under the MCS75. We analysed the data for the fiscal year (FY) 2018 (from April 2018 to March 2019), as this was the latest year of municipality-level data available.

Data on the municipality- and SMA-level explanatory variables were retrieved from various government survey results available on the websites of the MHLW, Ministry of Internal Affairs and Communication, and e-Stat (portal site of official statistics of Japan). The municipality-level explanatory variables are as follows:

1. Demographic data: proportion of the population aged 75 and above and workers in the primary industry.
2. Economic indices of the population: unemployment rate and taxable income per taxpayer.
3. Proxy for rurality: population density.
4. Health status indices: male and female life expectancy and the proportion of older adults (aged 65 and above) certified as needing long-term care.
5. Factors that could potentially offset hospitalisation costs: outpatient medical expenditure *per capita* and long-term care insurance benefit expenditure per recipient, which includes expenses for both in-home and facility services.

The indices of healthcare resources served as SMA-level variables. Since residents not only utilise medical facilities in their own municipalities but also those in neighbouring municipalities, the SMA-level indices were thought to provide a more accurate representation of the actual healthcare resources available in the locality. They included the following: number of doctors; number of hospital beds for general, psychiatric, and chronic care; average number of hospital stays (including all types of beds); number of home medical care visits by doctors in 1 month; and number of end-of-life care cases at home in 1 month, serving as an indicator of the healthcare infrastructure’s capacity to support end-of-life care at home. The SMA-level number of doctors was generated by aggregating the municipality-level data. Except for hospitalisation days, the number per 100,000 residents was calculated for these variables. Additionally, the RHWB regions were incorporated into the models as a prefecture-level explanatory variable. Details of the variable descriptions and their corresponding data sources are provided in Supplementary Table 2.

### Statistical analysis

In our bivariate analysis, we examined the relationship between the RHWB regions, IMEP75, and other explanatory variables using cross-tabulation. To statistically compare each variable across the seven RHWB regions, we performed Kruskal-Wallis tests, as all the variables were non-normally distributed. To visually represent geographic variation, we mapped IMEP75 across the municipalities with QGIS version 3.22.9, using a shapefile obtained from a government website (26), and constructed a box and whisker plot of IMEP75 across the seven RHWB regions, together with *per capita* outpatient expenditure.

For the multivariate analysis, we constructed multilevel linear regression models with three levels: municipalities, SMAs, and prefectures. Since the number of RHWB regions was considered insufficient to be incorporated into the models at the fourth level (27), we adopted a modelling approach similar to that presented by Adedini et al. (28), and used the RHWB regions as an explanatory variable at the prefecture level instead. The Kanto-Shinetsu region, with the largest number of municipalities, served as a reference.

We constructed random intercept models with fixed slopes, as follows:


$$y_{ijk}=\beta_{0}+\beta_{1}x_{1}+\ldots +v_{k}+u_{jk}+e_{ijk}$$

where *y_ijk_* denotes the medical expenditure for municipality *i* in SMA *j* in prefecture *k*; β_0_ is the overall mean or the constant; β_1_ is the coefficient for the explanatory variable *x_1_*; *v*_*k*,_ *u_jk,_* and *e_ijk_* are the residual error terms for the municipality-, SMA-, and prefecture-levels, respectively. *v*_*k*,_ *u_jk_* and *e_ijk_* collectively represent the random effects of the model, whereas the remaining terms explain its fixed effects. The coefficients for each explanatory variable were estimated with their 95% confidence intervals (CI) and value of ps, and the variances at each level were estimated using the standard error (SE).

To examine the effects of the explanatory variables on IMEP75 and the variances at the municipality, SMA, and prefecture levels, we fitted six models. Model 1 (empty model) included the three levels with no explanatory variables. Models 2, 3, and 4 exclusively incorporated the municipality-, SMA-, and prefecture-level variables. Model 5 considered the SMA- and prefecture-level variables, and Model 6 (full model) included all the variables. To determine the proportion of total variance that can be explained by each level, the variance partition coefficient (VPC) was calculated, as follows (29):


$$VPC_{v}=\frac{\sigma_{v}^{2}}{\sigma_{v}^{2}+\sigma_{u}^{2}+\sigma_{e}^{2}}$$

where the numerator denotes the variance at level *v* and the denominator is the sum of all variances at the three levels. Furthermore, to evaluate the extent to which the selected variables contributed to explaining the observed variances at each level, the proportional change in variance (PCV) was calculated using the following formula, with Model 1 as the reference (29):


$$PCV_{v}\;\left(\%\right)=100\times \frac{\sigma_{v\left[\mathrm{model}1\right]}^{2}-\sigma_{v\left[\mathrm{model}\;2\right]}^{2}}{\sigma_{v\left[\mathrm{model}1\right]}^{2}}$$

We assessed the goodness-of-fit of the constructed models by computing the Akaike Information Criterion (AIC) and Bayesian information Criterion (BIC). The multicollinearity between the selected variables was assessed by calculating the variance inflation factor.

The robustness of our results was tested using two additional sensitivity analyses. First, to verify the consistency of our findings across the two different time periods, we repeated the regression modelling using IMEP75 in FY 2017 as the outcome variable. Second, to fully account for the differences in population composition among municipalities, we used the Regional Disparity Index (RDI) of inpatient medical expenditures for FY 2018 as the outcome variable in the regression models. The RDI is the ratio of the observed municipality per-capita inpatient medical expenditure to the expected value calculated from the age group-specific national average of medical expenditure and the population composition of the municipality (30). An RDI value of 1 is expected if the medical expenditure of each age group in the municipality is the same as the national average. A value greater than 1 indicates higher medical expenditure for the municipality, while a value less than 1 suggests lower expenditure. We used RDI values that were also available in the MHLW dataset (9).

The statistical significance level was set at 0.05. All analyses were performed using STATA software (version 17.0; StataCorp LLC).

### Ethics statement

Given that we handled only publicly available aggregate data with no individual patient information, the study was deemed exempt from an institutional ethics review. It was reported in accordance with Strengthening the Reporting of Observation Studies in Epidemiology (STROBE) guidelines.

## Results

**Figure 1:**
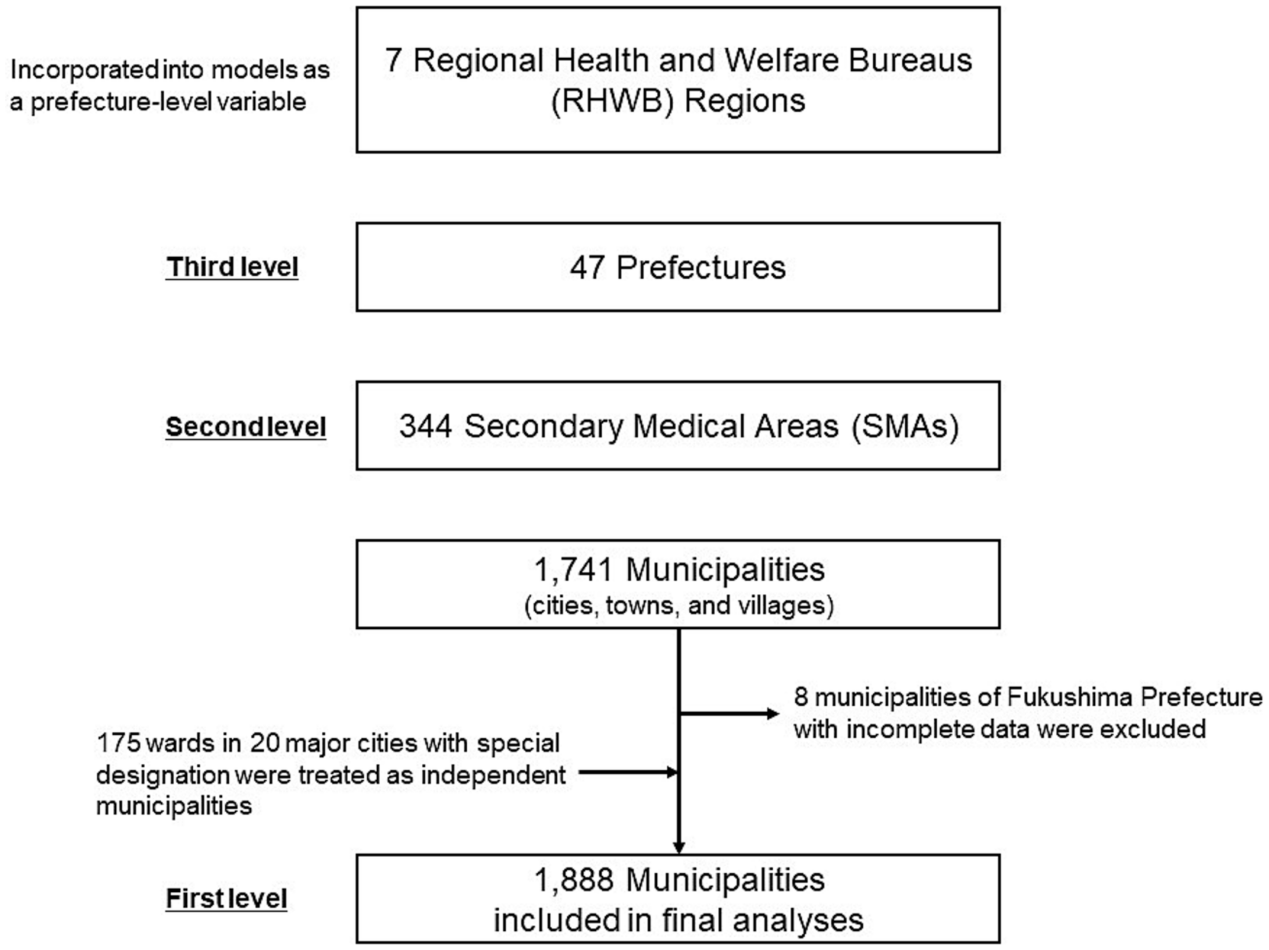
Overview of the geographic unit hierarchy and municipalities included in the final analyses.

After excluding eight municipalities in Fukushima Prefecture with incomplete demographic data (due to the Great East Japan Earthquake in 2011), a total of 1,888 municipalities, 344 SMAs, 47 prefectures, and seven RHWB regions were included in our final analysis (Figure 1).

Table 1 summarises the number and percentage of municipalities, SMAs, and prefectures, along with the means of IMEP75 and municipality- and SMA-level variables across the seven RHWB regions. Significant geographic differences were observed for all variables (*p* < 0.001). The mean IMEP75 was the highest in the Kyushu region (606 thousand JPY; Approximately 4,300 USD) and lowest in the Tohoku region (381 thousand JPY; Approximately, 2,700 USD) with a difference of 225 thousand JPY (approximately 1,600 USD). Regarding the municipality-level variables, the RHWB regions housing megapolitan areas such as Tokyo (Kanto-Shinetsu region), Nagoya (Tokai-Hokuriku region), and Osaka (Kinki region) exhibited lower proportions of the population aged 75 and above and workers in the primary industry. By contrast, these regions had a higher population density and taxable income per taxpayer. Notable disparities were observed in the healthcare delivery indices at the SMA-level. The number of hospital beds (per 100,000 people) for general, psychiatric, and chronic care, as well as the average length of hospital stay was higher in the Chugoku-Shikoku, Kyushu, and Hokkaido regions than in other regions. The number of doctors (per 100,000 people) was the highest in the Chugoku-Shikoku region (238) and lowest in Hokkaido (176). The number of home medical care visits by doctors (per 100,000 population in 1 month) was the highest in Chugoku-Shikoku (1030) and lowest in Tohoku (535), whereas the number of end-of-life care cases at home (per 100,000 people in 1 month) ranged from the lowest at 4 in the Hokkaido region to the highest at 10 in the Kinki region.

**Table 1:** Summary of the geographic units and municipality and secondary medical area-level variables across the seven regional health and welfare bureau regions, and the results of bivariate analyses by Kruskal-Wallis test (shown by value of ps).

	Regional health and welfare bureau regions								
	Overall	Hokkaido	Tohoku	Kanto-Shinetsu	Tokai-Hokuriku	Kinki	Chugoku-Shikoku	Kyushu	Value of *p*
**Geographic units**									
Municipalities: n (%)	1888	188 (10.0)	223 (11.8)	496 (26.3)	217 (11.5)	262 (13.9)	212 (11.2)	290 (15.4)	
Secondary medical areas: n (%)	344	21 (6.1)	38 (11.0)	89 (25.9)	37 (10.8)	47 (13.7)	48 (14.0)	64 (18.6)	
Prefectures: n (%)	47	1 (2.1)	6 (12.8)	10 (21.3)	6 (12.8)	7 (14.9)	9 (19.1)	8 (17.0)	
**Outcome variable**									
Inpatient medical care expenditure *per capita* in FY2018 (1,000JPY): mean (sd)	480 (114)	560 (121)	381 (67)	404 (55)	422 (67)	506 (65)	545 (99)	606 (90)	<0.001
**Explanatory variables**									
**Municipality-level variables (*n* = 1,888)**									
Proportion of population aged 75 years or over: % (sd)	17.0 (5.3)	19.2 (4.2)	18.8 (4.7)	15.2 (5.1)	15.1 (4.9)	16.0 (5.1)	20.1 (5.6)	17.1 (4.8)	<0.001
Proportion of workers in primary industry: % (sd)	10.2 (10.2)	21.4 (13.0)	14.2 (9.2)	6.9 (8.2)	4.6 (4.5)	4.8 (6.3)	12.6 (9.4)	12.7 (10.4)	<0.001
Unemployment rate: % (sd)	4.1 (1.3)	3.3 (1.6)	4.2 (1.2)	3.9 (1.0)	3.4 (0.8)	4.5 (1.2)	3.9 (1.2)	4.7 (1.7)	<0.001
Taxable income per taxpayer (100,000JPY): mean (sd)	29 (5)	28 (5)	25 (3)	31 (7)	30 (4)	30 (4)	26 (3)	26 (3)	<0.001
Population density (10people/km^2^): mean (sd)	154 (321)	26 (93)	22 (45)	275 (445)	152 (217)	305 (443)	45 (97)	79 (155)	<0.001
Male life expectancy (years): mean (sd)	80.6 (0.8)	80.3 (0.5)	79.9 (0.9)	80.9 (0.7)	81 (0.5)	80.8 (1.0)	80.5 (0.6)	80.5 (0.7)	<0.001
Female life expectancy (years): mean (sd)	87.0 (0.6)	86.8 (0.4)	86.6 (0.6)	87.0 (0.6)	87.0 (0.4)	87.0 (0.6)	87.2 (0.5)	87.2 (0.5)	<0.001
Proportion of older adults certified as needing long-term care: % (sd)	13.4 (2.3)	13.6 (1.6)	14.7 (2.0)	12.6 (2.1)	11.8 (2.8)	13.8 (2.1)	14.7 (1.9)	13.8 (1.8)	<0.001
Long-term care benefit expenditure per recipient in FY2018 (1,000JPY): mean (sd)	1,618 (199)	1,422 (199)	1,688 (194)	1,654 (195)	1,672 (144)	1,506 (168)	1,628 (147)	1,680 (184)	<0.001
Outpatient medical care expenditure *per capita* in FY2018 (1,000JPY): mean (sd)	399 (52)	404 (45)	375 (39)	386 (48)	411 (42)	424 (53)	409 (52)	398 (60)	<0.001
**Secondary medical area-level variables (*n* = 344)**									
Number of hospital beds for general care (per 100,000 population): mean (sd)	731 (223)	915 (233)	754 (211)	630 (198)	599 (179)	718 (170)	823 (167)	814 (250)	<0.001
Number of hospital beds for psychiatric care (per 100,000 population): mean (sd)	311 (208)	409 (324)	318 (119)	220 (151)	208 (130)	215 (137)	378 (173)	483 (234)	<0.001
Number of hospital beds for chronic care (per 100,000 population): mean (sd)	313 (202)	486 (272)	185 (114)	205 (126)	277 (141)	252 (116)	464 (238)	434 (181)	<0.001
Average number of days in hospital for all types of hospital beds combined: mean (sd)	34 (13)	38 (14)	30 (7)	28 (9)	30 (10)	30 (8)	37 (11)	44 (16)	<0.001
Number of doctors (per 100,000 population): mean (sd)	212 (98)	176 (65)	178 (60)	214 (144)	197 (68)	226 (75)	238 (78)	221 (82)	<0.001
Number of home visiting care by doctor (visits per 100,000 population in one month): mean (sd)	789 (427)	592 (411)	535 (315)	789 (402)	696 (263)	842 (386)	1,030 (534)	841 (435)	<0.001
Number of end-of-life care at home (cases per 100,000 population in one month): mean (sd)	8 (5)	4 (3)	9 (6)	10 (5)	9 (4)	10 (4)	8 (4)	7 (4)	<0.001

**Figure 2:**
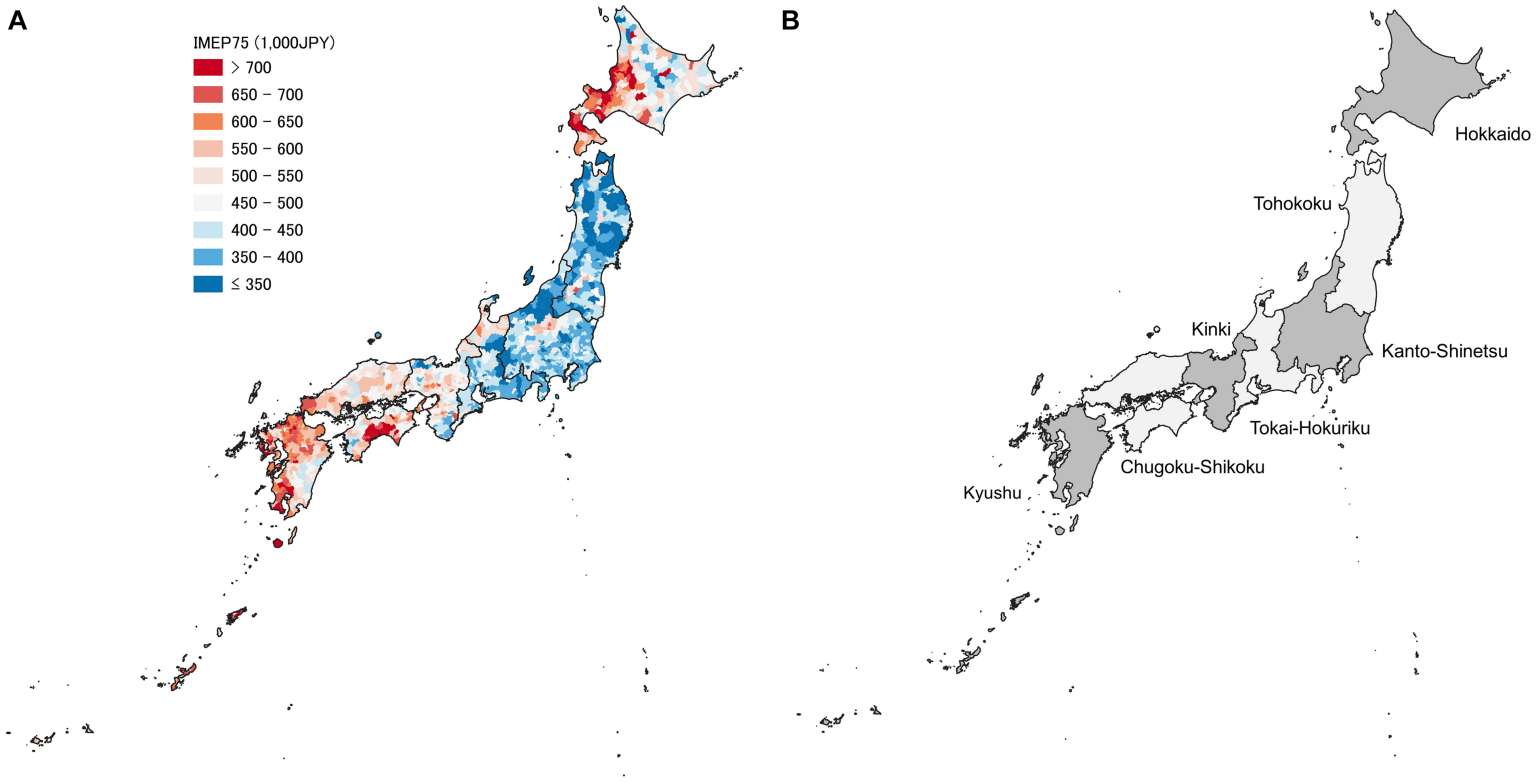
Map of FY 2018 inpatient medical expenditure *per capita* for older adults aged 75 and above (IMEP75) across the municipalities in Japan **(A)**. The grey shade represents the categorical range encompassing the overall average value of 480 thousand JPY. The seven regional health and welfare bureau regions are also shown **(B)**.

**Figure 3:**
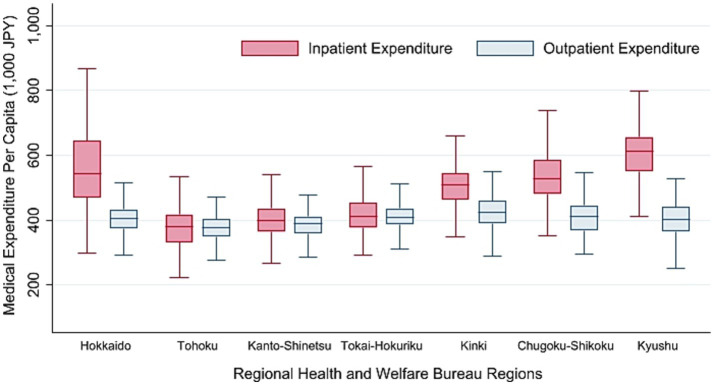
Box and Wisker plot of FY 2018 inpatient and outpatient medical expenditure *per capita* for older adults aged 75 and above across the seven regional health and welfare bureau regions.

Figure 2 graphically represents the geographic distribution of IMEP75 across the municipalities and locations of the seven RHWB regions. Figure 3 shows a box and whisker plot of IMEP75 and *per capita* outpatient expenditure across the RHWB regions. Notably, regional disparities in IMEP75 were appreciably larger than those in *per capita* outpatient expenditure.

Table 2 presents the results of the multilevel linear regression analyses. In Model 1 (empty model), the VPC showed that 27, 15, and 57% of the observed total variance were present at the municipality-, SMA-, and prefecture-levels, respectively. As indicated by the PCV, addition of the municipality-level variables in Model 2 resulted in a decrease of 5.6, 13.2, and 7.5% in the variances at the municipality-, SMA-, and prefecture-levels, respectively, and of 7.8% in the total variance. By incorporating the SMA-level variables in Model 3, we observed a large decrease of 43.1, 46.8, and 33.5% in the SMA-level, prefecture-level, and total variances, respectively, suggesting that the differences in the availability of healthcare resources could explain a substantial portion of the observed between-SMA, between-prefecture, and total variances. Model 4, which incorporated the RHWB regions, led to a significant reduction of 83.7 and 47.9% in the prefecture-level and total variances, respectively, stating that the RHWB regions were accountable for the vast majority of the observed variance at the prefecture level. Combination of the SMA-level variables and the RHWB regions in Model 5 decreased the SMA- and prefecture-level variances by 42.8 and 88.4%, respectively, and the total variance by 57.3%. Inclusion of all the variables in Model 6 (final model) explained 5.8, 53.1, and 87.3% of the municipality-, SMA-, and prefecture-level variances, respectively, and 59.8% of the total variance. The VPCs were now 0.64, 0.18, and 0.18 for the municipality-, SMA-, and prefecture-levels, respectively, showing an appreciable shift in the distribution of the VPC from the null model, with the majority of the total variance concentrated at the municipality-level.

**Table 2:** Multilevel linear regression models of inpatient medical care expenditure *per capita* (fy 2018) for adults aged 75 years and above in association with municipality-, secondary medical area-, and prefecture-level variables.

	Model 1			Model 2			Model 3		
	Empty model			Municipality-level variables			Secondary medical area-level variables		
Fixed effects	B	95% CI	Value of *p*	B	95% CI	Value of *p*	B	95% CI	Value of *p*
**Municipality-level variables**									
Proportion of population aged 75 years or above				−0.55	(−1.5, 0.4)	0.26			
Proportion of workers in primary industry				−0.54	(−1.01, −0.08)	0.02			
Unemployment rate				−0.82	(−3.88, 2.25)	0.60			
Population density				−0.02	(−0.04, −0.002)	0.03			
Taxable income per tax payer				−0.52	(−1.47, 0.43)	0.28			
Male life expectancy				−7.92	(−14.8, −1.04)	0.02			
Female life expectancy				10.26	(1.69, 18.82)	0.02			
Proportion of older adults certified as needing long-term care				5.80	(3.81, 7.78)	<0.001			
Long-term care benefit expenditure				−0.08	(−0.10, −0.06)	<0.001			
Outpatient medical care expenditure				0.13	(0.05, 0.21)	0.002			
**Secondary medical area-level variables**									
Number of hospital beds for general care							0.03	(0.002, 0.06)	0.04
Number of hospital beds for psychiatric care							0.06	(0.03, 0.09)	0.001
Number of hospital beds for chronic care							0.07	(0.03, 0.11)	0.001
Average number of days in hospital							0.97	(0.20, 1.74)	0.01
Number of doctors							0.11	(0.04, 0.17)	0.002
Number of home visiting care by doctor							0.02	(0.004, 0.04)	0.01
Number of end-of-life care at home							−2.17	(−3.50, −0.84)	0.001
**Prefecture-level variable (RHWB regions)**									
(Reference: Kanto-Shinetsu)									
Hokkaido									
Tohoku									
Tokai-Hokuriku									
Kinki									
Chugoku-Shikoku									
Kyushu									
Random effects	Model 1 (Empty model)			Model 2 (Municipality)			Model 3 (SMA)		
**Municipality-level**									
Variance (SE)	3,324	(119)		3,139	(112)		3,310	(118)	
VPC (Variance Partition Coefficient)	0.27			0.28			0.41		
Explained variance: % (i.e., Proportional Change in Variance)	Ref.			5.6			0.4		
**Secondary medical area-level**									
Variance (SE)	1844	(210)		1,600	(189)		1,049	(141)	
VPC	0.15			0.14			0.13		
Explained variance: %	Ref.			13.2			43.1		
**Prefecture-level**									
Variance (SE)	6,955	(1516)		6,435	(1410)		3,697	(848)	
VPC	0.57			0.58			0.46		
Explained variance: %	Ref.			7.5			46.8		
**Total**									
Variance (sum of three levels)	12,123			11,174			8,056		
Explained variance: %	Ref.			7.8			33.5		
**Model fit statistics**									
Log-likelihood	−10,625			−10,562			−10,554		
AIC	21,258			21,151			21,130		
BIC	21,280			21,229			21,191		
	Model 4			Model 5			Model 6		
	Prefecture-level variable			Secondary medical-area and prefecture-level variables			Full model with all variables		
Fixed effects	B	95% CI	Value of *p*	B	95% CI	Value of *p*	B	95% CI	Value of *p*
**Municipality-level variables**									
Proportion of population aged 75 years or above							−0.92	(−1.85, 0.005)	0.051
Proportion of workers in primary industry							−0.58	(−1.03, −0.13)	0.01
Unemployment rate							−2.26	(−5.24, 0.73)	0.14
Population density							−0.01	(−0.03, 0.005)	0.16
Taxable income per tax payer							−0.65	(−1.60, 0.30)	0.18
Male life expectancy							−8.40	(−15.06, −1.74)	0.01
Female life expectancy							10.38	(2.04, 18.72)	0.02
Proportion of older adults certified as needing long-term care							5.71	(3.77, 7.65)	<0.001
Long-term care benefit expenditure							−0.08	(−0.10, −0.06)	<0.001
Outpatient medical care expenditure							0.12	(0.04, 0.20)	0.004
**Secondary medical area-level variables**									
Number of hospital beds for general care				0.03	(<0.001, 0.06)	0.046	0.03	(−0.001, 0.06)	0.06
Number of hospital beds for psychiatric care				0.06	(0.02, 0.09)	0.001	0.05	(0.02, 0.08)	0.002
Number of hospital beds for chronic care				0.07	(0.03, 0.11)	<0.001	0.07	(0.03, 0.11)	<0.001
Average number of days in hospital				0.80	(0.03, 1.56)	0.04	0.92	(0.20, 1.63)	0.01
Number of doctors				0.11	(0.04, 0.17)	0.002	0.11	(0.04, 0.17)	0.001
Number of home visiting care by doctor				0.02	(0.001, 0.03)	0.03	0.01	(−0.01, 0.02)	0.34
Number of end-of-life care at home				−2.05	(−3.36, −0.74)	0.002	−1.79	(−3.04, −0.53)	0.005
**Prefecture-level variable (RHWB regions)**									
(Reference: Kanto-Shinetsu)									
Hokkaido	155.96	(82.86, 229.06)	<0.001	103.39	(40.62, 166.16)	0.001	86.36	(21.09, 151.64)	0.01
Tohoku	−30.13	(−69.63, 9.37)	0.135	−34.03	(−67.56, −0.49)	0.047	−43.59	(−78.32, −8.85)	0.01
Tokai-Hokuriku	32.48	(−7.38, 72.35)	0.110	23.74	(−9.77, 57.25)	0.17	21.01	(−13.28, 55.29)	0.23
Kinki	91.10	(53.49, 128.70)	<0.001	81.39	(49.70, 113.08)	<0.001	63.65	(31.12, 96.18)	<0.001
Chugoku-Shikoku	131.93	(96.11, 167.75)	<0.001	80.41	(48.73, 112.1)	<0.001	66.95	(34.35, 99.56)	<0.001
Kyushu	186.99	(151.08, 222.90)	<0.001	130.40	(98.69, 162.11)	<0.001	127.85	(95.19, 160.51)	<0.001
Random effects	Model 4 (Prefecture)			Model 5 (SMA & Prefecture)			Model 6 (Full model)		
**Municipality-level**									
Variance (SE)	3,324	(119)		3,310	(118)		3,132	(112)	
VPC (Variance Partition Coefficient)	0.53			0.64			0.64		
Explained variance: % (i.e., Proportional Change in Variance)	0.0			0.4			5.8		
**Secondary medical area-level**									
Variance (SE)	1858	(212)		1,054	(142)		865	(126)	
VPC	0.29			0.20			0.18		
Explained variance: %	−0.8			42.8			53.1		
**Prefecture-level variance**									
Variance (SE)	1,137	(333)		807	(236)		881	(244)	
VPC	0.18			0.16			0.18		
Explained variance: %	83.7			88.4			87.3		
**Total**									
Variance (sum of three levels)	6,319			5,171			4,878		
Explained variance: %	47.9			57.3			59.8		
**Model fit statistics**									
Log-likelihood	−10,589			−10,524			−10,462		
AIC	21,199			21,081			20,978		
BIC	21,254			21,176			21,127		

B, unstandardized beta coefficient; CI, confidence interval.

Table 2 also shows the effect of each explanatory variable on the outcomes of the fixed effects of the models. In the full model, a higher proportion of workers in the primary industry was associated with a lower IMEP75. The proportion of population aged 75 and above showed a similar trend. A higher proportion of older adults certified as needing long-term care and *per capita* outpatient medical expenditure increased IMEP75, whereas a higher long-term care benefit expenditure per recipient decreased IMEP75. Additionally, male and female life expectancies exerted divergent effects on the outcomes. Specifically, higher male life expectancies were associated with lower IMEP75 levels, whereas the opposite was observed for female life expectancies.

With regard to the SMA-level characteristics, a higher number of doctors, hospital beds for psychiatric and chronic care (but not for general care), and average hospital stay, were all associated with a higher IMEP75. Conversely, IMEP75 decreased with a higher number of end-of-life care cases at home. Finally, compared to the Kanto-Shinetsu region, IMEP75 was significantly higher in the Hokkaido, Kinki, Chugoku-Shikoku, and Kyushu regions, and lower in the Tohoku region. Notably, the coefficients for some of the RHWB regions decreased between Models 4 and 5, and 5 and 6, suggesting that the SMA- and municipality-level variables were partly accountable for regional differences. The possibility of multicollinearity among the selected variables was deemed unlikely, because the variance inflation factors were all below five (Supplementary Table 3). A comparison of the AIC and BIC values among the six models consistently favoured the full model as the best fit for the data.

### Sensitivity analysis results

Supplementary Tables 4, 5 present the results of the sensitivity analyses using the FY 2017 IMEP75 and FY 2018 RDI as the outcome variables, respectively. We observed similar patterns of changes in the municipality-level, SMA-level, prefecture-level, and total variances across the six models. Associations between the individual explanatory variables and the outcome displayed consistent patterns, although a higher number of general hospital beds was associated with a higher IMEP75, and the association between the proportion of workers in the primary industry did not reach statistical significance in FY2017.

## Discussion

In this multilevel analysis of nationwide data, we found that healthcare resources in the SMA-level and RHWB regions explained a large proportion of the overall geographic variation in IMEP75, whereas the effects of municipality-level characteristics were relatively small. Additionally, we identified several factors associated with IMEP75.

Evaluation of the PCVs across the six models consistently indicated that the availability (i.e., supply) of medical services at the SMA-level was a more important driver of geographic variation in IMEP75 in Japan than factors related to healthcare needs (i.e., demand) at the municipality-level. As suggested by previous reports from Japan (9, 10, 31), we observed a higher IMEP75 with an increasing number of doctors, hospitalisation days, and psychiatric and chronic care beds. Similarly, a US study reported that post-acute care such as the use of long-term care hospitals, hospices, home care services, and nursing and rehabilitation facilities accounted for a large portion of the geographic variation in Medicare expenditure (32). Healthcare utilisation and costs are known to increase sharply during the last year of life (33, 34), owing largely to an increased disease and care burden resulting from the presence of multiple chronic conditions and functional impairment, including dementia (35). Furthermore, hospitals are the primary place for end-of-life care in Japan, with 72% of all deaths occurring in hospitals in 2018 (36). In light of this evidence, our results imply that in areas with excess psychiatric and chronic care beds, they are more likely to be utilised for the care of older adults with dementia and/or chronic conditions, thereby assuming the function of long-term care facilities and end-of-life care provision. Notably, we observed a lower IMEP75 with an increasing number of end-of-life care cases at home, whereas the number of home visits by doctors did not significantly affect IMEP75. This emphasises the importance of establishing a comprehensive home-visit care system that can support patients throughout their final stages of life to effectively mitigate the burden on inpatient care utilisation.

Although the largest portion of the overall geographic variation in IMEP75 was evident at the prefecture-level (57%), the vast majority (83.7%) of this between-prefecture variation was explained solely by the RHWB regions. Consistent with a previous report (9), IMEP75 was high in the North (Hokkaido region) and West (Kinki, Chugoku-Shikoku, and Kyushu regions), and low in the East (Tohoku region). Despite accounting for the municipality- and SMA-level correlates, the RHWB regions remained significant determinants of IMEP75, indicating that unobserved factors specific to these regions also contributed to regional disparities. Potential factors include differences in the number and capacity of tertiary medical centres offering advanced care, hospital ownership (37), availability of specialist physicians (12), prevailing standard clinical practices in the locality (38), and patient and family preferences shaped by sociocultural factors (18, 39). All of these may influence the aggressiveness of diagnostic testing, selection of treatment modalities, and intensity of treatment, thereby affecting the average unit cost of hospitalisation. Further research is needed to explore the underlying causes of disparities between the RHWB regions.

Certain municipality-level demographic factors also influenced IMEP75. A higher proportion of workers in the primary industry was associated with a lower IMEP75, and a higher proportion of people aged 75 and above showed a non-significant trend of decreasing IMEP75. While those in the primary industry may exhibit unique healthcare utilisation patterns or enjoy better health status from their lifestyle, the scarcity of healthcare resources could also contribute to the observed associations, as municipalities with higher proportions of primary industry workers and advanced population ageing are generally located in rural areas. Additionally, IMEP75 increased with the proportion of older adults certified as needing long-term care. This finding justifies the Japanese government’s ongoing zealous initiatives to prevent frailty and long-term care needs of older adults through primary and secondary disease prevention and community-based measures such as organising exercise programmes and promoting social participation (40).

We considered two municipality-level factors that could potentially offset IMEP75. Higher long-term benefit expenses per recipient, which served as a proxy for the availability of long-term care resources, were associated with lower IMEP75. This suggests that establishing a robust system of long-term care provision for older adults outside hospitals could contribute to curbing IMEP75. By contrast, we observed a higher IMEP75 with increasing *per capita* outpatient medical expenditure. Overseas studies have demonstrated the role of adequate and continued primary care provision in preventing hospitalizations for ambulatory care-sensitive conditions (41–43). However, in the context of Japan, where the surplus of psychiatric and chronic care beds seems to be a key driver for prolonging hospitalisation and increasing IMEP75, spending on outpatient care may contribute little to reducing IMEP75. Furthermore, while more primary care visits in the preceding year is linked to reduced end-of-life hospital utilisation and costs in the US (44), the low adoption of advance directives in Japan may be associated with avoidable emergency transfers and hospital admissions (45–47).

Finally, longer male and female life expectancies were associated with lower and higher IMEP75 levels, respectively. A cross-country analysis of OECD nations found that an increase in healthcare spending was associated with greater life expectancy gains for men, except in Japan, where gains were greater for women (48) – a finding in line with ours. Since sex-specific data on IMEP75 were unavailable, further analysis stratified by sex was not possible. However, it is reasonable to consider that men and women may experience different health conditions, care needs, and caregiving capabilities of families at home during the later stages of life, all of which can affect inpatient care utilisation. Further research is warranted to elucidate the underlying mechanisms.

### Strengths and limitations

The primary strength of our study lies in its analytical design. We used the aggregate insurance claims data from the MCS75 dataset, which covered virtually all older adults aged 75 and above in Japan. Excluding only eight municipalities from the analytical sample, our results provide a comprehensive overview of the entire country. Further, to the best of our knowledge, this is the first study to employ a multilevel analytical approach to effectively quantify the magnitude of geographic variations in IMEP75 at the three administrative levels in Japan and reveal the factors contributing to them.

This study has several limitations. First, we used indices of healthcare provision at the SMA-level. However, these indicators may not fully reflect the healthcare resources utilised by residents in each municipality. For instance, in some municipalities, residents may solely utilise healthcare facilities within their own municipality, whereas in others, they may rely predominantly on healthcare facilities in an adjacent SMA. This may have led to information bias by linking municipalities with inappropriate healthcare resource data.

Second, as discussed earlier, our analyses did not adequately address the mechanism of geographic disparities at the RHWB region-level and explained only a small portion of the variations in IMEP75 at the municipality-level. This suggests the presence of other important municipality-level factors not considered in our analyses. Municipality-level data on health indicators, such as smoking rate, prevalence of specific medical conditions, health check-up attendance rate, and healthy life expectancy, were not publicly available for inclusion in our analyses; therefore, we may not have adequately captured population health. Healthcare accessibility might also play an important role in creating disparities between municipalities (49). With mountainous areas making up approximately 75% of Japan’s land area (50), the accessibility to healthcare services can vary significantly, even within the same SMA (51). Due to the lack of specific indices of rurality in our dataset, we opted to use population density as a proxy. Nevertheless, we may not have sufficiently explored urban/rural differences and geographical disadvantages in transport. To gain insight into the impact of accessibility on healthcare utilisation and costs, factors such as additional measure of rurality, hospital density and travel time to hospitals should be considered in future analyses.

Third, our study design does not permit assessing the quality and efficiency of medical care delivery owing to geographic differences in healthcare costs. Some evidence has indicated that increased spending on regional healthcare does not necessarily correlate with improved outcomes (52, 53). With a consensus on the optimal level of regional healthcare spending for older adults’ care yet to be established in Japan, further studies are required to develop goal-oriented healthcare spending guided by rational quality evaluation indicators.

Finally, caution should be exercised when generalising our findings to other countries because our data are specific to Japan’s healthcare and medical insurance systems.

### Policy implications

Despite these limitations, our results have important policy implications for other countries as they provide valuable evidence from Japan—a country at the forefront of population ageing that can inform the design of healthcare systems for the older population. First, this study emphasises the importance of incorporating small- and large-area analyses of healthcare cost disparities to develop a comprehensive understanding of underlying mechanisms and appropriate interventions. In the case of Japan, our findings suggest that focusing solely on the reorganisation and optimisation of healthcare resources within SMAs, as currently promoted under the “Regional Healthcare Planning,” is unlikely to fully resolve the geographic disparities in IMEP75. This is because discussions within a confined area will not necessarily motivate the reallocation of healthcare resources across SMAs, prefectures, or RHBW regions. Interventions are required that specifically target disparities in larger areas. Second, to effectively address the rising inpatient costs for older adults and the associated geographic disparities, healthcare delivery must integrate the design of a long-term care system with an end-of-life care infrastructure that adequately supports older adults outside of hospitals.

## Conclusion

We found that the availability of medical services and RHWB regions, rather than factors relevant to healthcare needs, were the most important determinants of geographic variation in IMEP75 in Japan. Future initiatives should prioritise the reallocation of medical resources outside the SMAs and evaluate the root causes of the geographic disparities in IMEP75 between the RHWB regions.

## Data availability statement

All raw data are accessible via government websites, the details of which are provided in the Supplementary material. The dataset used for our analyses is available upon reasonable request by researchers to the corresponding author, after we have completed planned analyses.

## Ethics statement

Ethical approval was not required for the study involving humans in accordance with the local legislation and institutional requirements. Written informed consent to participate in this study was not required from the participants or the participants’ legal guardians/next of kin in accordance with the national legislation and the institutional requirements.

## Author contributions

YSK: Conceptualization, Data curation, Formal analysis, Investigation, Methodology, Visualization, Writing – original draft, Writing – review & editing. YSG: Formal analysis, Investigation, Methodology, Supervision, Validation, Writing – review & editing. RS: Supervision, Writing – review & editing.
