# Supplementary material for: Geographic variation in inpatient medical expenditure among older adults aged 75 years and above in Japan: a three-level multilevel analysis of nationwide data
Source: Front Public Health. 2024 Feb 28;12:1306013. doi: 10.3389/fpubh.2024.1306013 (PMC10933056; doi:10.3389/fpubh.2024.1306013)
Supplement: Supplementary file 1 [file Data_Sheet_1.docx]

**Supplementary Materials**

**TABLE OF CONTENTS**

**Supplementary Table 1.** Summary of Regional Health and Welfare Bureau (RHWB) Regions, Respective Prefectures within Each Region, Regional Populations, and Nominal Gross Regional Products in Year 2018.2

**Supplementary Table 2.** Details of Variable Descriptions and Data Sources.3

**Supplementary Table 3.** Variance Inflation Factors (VIF) for Explanatory Variables. 5

**Supplementary Table 4.** Multilevel Linear Regression Models of Inpatient Medical Expenditure Per Capita (FY 2017) for Older Adults Aged 75 Years and Above in Association with Municipality-, Secondary Medical Area-, and Prefecture-level Variables. 6

**Supplementary Table 5.** Multilevel Linear Regression Models of Regional Disparity Index of Inpatient Medical Expenditure (FY 2018) for Older Adults Aged 75 Years and Above in Association with Municipality-, Secondary Medical Area-, and Prefecture-level Variables. 8

**Supplementary References.** 10

**Supplementary Table 1. Summary of Regional Health and Welfare Bureau (RHWB) Regions, Respective Prefectures within Each Region, Regional Populations, and Nominal Gross Regional Products in Year 2018.**

| **RHWB regions** | **Prefectures** | **Regional population [1] (proportion to national population)** | **Gross regional product^†^: 1 million JPY [2]  (proportion to GDP)** | **Gross regional product^‡^ per capita:  1 million JPY** |
| --- | --- | --- | --- | --- |
| **Hokkaido** | Hokkaido | 5,339,539 | 20,431,461 | 3.83 |
|  |  | (4.2%) | (3.5%) |  |
| **Tohoku** | Aomori, Iwate, Miyagi, Akita, Yamagata, Fukushima | 8,926,837 | 35,346,178 | 3.96 |
|  |  | (7.0%) | (6.0%) |  |
| **Kanto-Shinetsu** | Ibaraki, Tochigi, Gunma, Saitama, Chiba, Tokyo, Kanagawa, Niigata, Yamanashi, Nagano | 48,632,286 | 250,280,392 | 5.15 |
|  |  | (38.1%) | (42.8%) |  |
| **Tokai-Hokuriku** | Toyama, Ishikawa, Gifu, Shizuoka, Aichi, Mie | 17,403,383 | 86,824,775 | 4.99 |
|  |  | (13.6%) | (14.9%) |  |
| **Kinki** | Fukui, Shiga, Kyoto, Osaka, Hyogo, Nara, Wakayama | 21,566,471 | 92,904,773 | 4.31 |
|  |  | (16.9%) | (15.9%) |  |
| **Chugoku-Shikoku** | Tottori, Shimane, Okayama, Hiroshima, Yamaguchi, Tokushima, Kagawa, Ehime, Kochi | 11,297,921 | 45,923,021 | 4.06 |
|  |  | (8.8%) | (7.9%) |  |
| **Kyushu** | Fukuoka, Saga, Nagasaki, Kumamoto, Oita, Miyagi, Kagoshima, Okinawa | 14,540,822 | 52,820,710 | 3.63 |
|  |  | (11.4%) | (9.0%) |  |
| ^†^Sum of gross prefectural products  ^‡^Gross regional product divided by regional population | | National population: 127,707,259 | GDP: 584,531,310 | GDP per capita: 4.58 |
|  |  |  |  |  |

**Supplementary Table 2. Details of Variable Descriptions and Data Sources.**

| **Variables** | **Data source** | **Year** | **Explanation** |
| --- | --- | --- | --- |
| **Outcome variables** | | | |
| Inpatient medical expenditure per capita for older adults aged 75 and above: 1,000 JPY | Analysis of Regional Differences in Medical Care Expenditures [3] | 2017 & 2018 |  |
| Regional disparity index (RDI) for inpatient medical expenditure |  | 2018 | The values generated by multiplying RDI by 100 were used in the analyses. |
| **Municipality-level explanatory variables** | | | |
| Proportion of population aged 75 years or over: % | Basic Resident Register: Population and Households [1] | 2017 & 2018 | Population aged 75 years and above divided by total population. |
| Proportion of workers in primary industry: % | 2015 Population Census [4] | 2015 | Number of workers in primary industry divided by total number of workers in all industries. |
| Unemployment rate: % | 2015 Population Census [4] | 2015 | Number of unemployed people divided by labour force population. |
| Population density: 10 people/km^2^ | 2015 Population Census [4] | 2015 |  |
| Taxable income per taxpayer: 100,000 JPY | Municipality taxation status [5] | 2015 | Total taxable income divided by number of taxpayers.  For those wards in the 20 major cities that were treated as individual municipalities in the analyses, city-level data were used for this variable, since ward-level data were unavailable. |
| Male and female life expectancy | Overview of Municipality Life Tables [6] | 2015 |  |
| Proportion of older people certified as needing long-term care | Long-Term Care Insurance Business Status Report [7] | 2017 & 2018 | Number of older adults certified as needing long-term care divided by population aged 65 years and above**^†^**. |
| Long-term care benefit expenditure per recipient: 1,000 JPY | Long-Term Care Insurance Business Status Report [7] | 2017 & 2018 | Total long-term care benefit expenditure divided by number of recipients aged 65 years and above**^†^**.  **^†^**The insurers of long-term care insurance　are　either　individual　municipalities　or　unions　consisting　of　two　to　several　neighbouring municipalities. The values for the unions were used in the analyses when applicable. |
| Outpatient medical expenditure per capita for older adults aged 75 and above: 1,000 JPY | Analysis of Regional Differences in Medical Care Expenditures [3] | 2017 & 2018 |  |

**Supplementary Table 2. continued**

| **Variables** | **Data source** | **Year** | **Explanation** |
| --- | --- | --- | --- |
| **Secondary medical area-level explanatory variables** | | | |
| Number of doctors per 100,000 people | Statistics of Physicians, Dentists, and Pharmacists [8] | 2016 | SMA-level numbers of doctors were generated by aggregating the municipality-level data.  Numbers per 100,000 people were calculated using secondary medical area populations |
| Number of hospital beds for general care per 100,000 people | Hospital Report [9] | 2017 | Numbers per 100,000 people were calculated using secondary medical area populations. |
| Number of hospital beds for psychiatric care per 100,000 people |  |  |  |
| Number of hospital beds for chronic care per 100,000 people |  |  |  |
| Number of home medical care visits by doctors in one month per 100,000 people | Survey of Medical Institutions [10] | 2017 |  |
| Number of end-of-life care cases at home in one month per 100,000 people |  |  |  |
| Average number of days in hospital (including all types of beds) | Hospital Report [9] | 2017 |  |

**Supplementary Table 3. Variance Inflation Factors (VIF) for Explanatory Variables.**

| **Explanatory Variables** | **VIF** |
| --- | --- |
| **Municipality-level variables** |  |
| Proportion of population aged 75 years or above | 2.69 |
| Proportion of workers in primary industry | 2.50 |
| Unemployment rate | 1.71 |
| Population density | 2.29 |
| Taxable income per tax payer | 2.89 |
| Male life expectancy | 2.99 |
| Female life expectancy | 2.49 |
| Proportion of older adults certified as needing long-term care | 1.88 |
| Long-term care benefit expenditure | 1.58 |
| Outpatient medical care expenditure | 1.76 |
| **Secondary medical area-level variables** |  |
| Number of hospital beds for general care | 2.18 |
| Number of hospital beds for psychiatric care | 2.59 |
| Number of hospital beds for chronic care | 3.01 |
| Average number of days in hospital | 4.08 |
| Number of doctors | 2.50 |
| Number of home visiting care by doctor | 2.07 |
| Number of end-of-life care at home | 1.63 |
| **Prefecture-level variable (RHWB regions)** |  |
| (Reference: Kanto-Shinetsu) |  |
| Hokkaido | 2.70 |
| Tohoku | 1.82 |
| Tokai-Hokuriku | 1.44 |
| Kinki | 1.66 |
| Chugoku-Shikoku | 2.20 |
| Kyushu | 2.37 |
| Mean VIF | 2.31 |

**Supplementary Table 4. Multilevel Linear Regression Models of Inpatient Medical Expenditure Per Capita (FY 2017) for Adults Aged 75 Years and Above in Association with Municipality-, Secondary Medical Area- and Prefecture-level Variables.**

|  | **Model 1** | | | **Model 2** | | | **Model 3** | | |
| --- | --- | --- | --- | --- | --- | --- | --- | --- | --- |
|  | **Empty model** | | | **Municipality-level variables** | | | **Secondary medical area-level variables** | | |
| **Fixed effects** | **B** | **95% CI** | **p-value** | **B** | **95% CI** | **p-value** | **B** | **95% CI** | **p-value** |
| **Municipality-level variables** |  |  |  |  |  |  |  |  |  |
| Proportion of population aged 75 years or above |  |  |  | -1.42 | (-2.37, -0.48) | 0.003 |  |  |  |
| Proportion of workers in primary industry |  |  |  | -0.31 | (-0.79, 0.16) | 0.19 |  |  |  |
| Unemployment rate |  |  |  | 1.25 | (-1.85, 4.36) | 0.43 |  |  |  |
| Population density |  |  |  | -0.02 | (-0.04, 0.004) | 0.02 |  |  |  |
| Taxable income per tax payer |  |  |  | -0.20 | (-1.16, 0.76) | 0.68 |  |  |  |
| Male life expectancy |  |  |  | -8.56 | (-15.53, -1.60) | 0.02 |  |  |  |
| Female life expectancy |  |  |  | 10.85 | (2.17, 19.52) | 0.01 |  |  |  |
| Proportion of older adults certified as needing long-term care |  |  |  | 6.01 | (4.10, 7.92) | <0.001 |  |  |  |
| Long-term care benefit expenditure |  |  |  | -0.06 | (-0.08, -0.04) | <0.001 |  |  |  |
| Outpatient medical care expenditure |  |  |  | 0.17 | (0.09, 0.25) | <0.001 |  |  |  |
| **Secondary medical area-level variables** |  |  |  |  |  |  |  |  |  |
| Number of hospital beds for general care |  |  |  |  |  |  | 0.04 | (0.005, 0.07) | 0.02 |
| Number of hospital beds for psychiatric care |  |  |  |  |  |  | 0.06 | (0.02, 0.09) | 0.001 |
| Number of hospital beds for chronic care |  |  |  |  |  |  | 0.08 | (0.04, 0.12) | <0.001 |
| Average number of days in hospital |  |  |  |  |  |  | 0.92 | (0.14, 1.71) | 0.02 |
| Number of doctors |  |  |  |  |  |  | 0.09 | (0.02, 0.16) | 0.009 |
| Number of home visiting care by doctor |  |  |  |  |  |  | 0.02 | (0.01, 0.04) | 0.003 |
| Number of end-of-life care at home |  |  |  |  |  |  | -2.22 | (-3.57, -0.87) | 0.001 |
| **Prefecture-level variable (RHWB regions)** |  |  |  |  |  |  |  |  |  |
| (Reference: Kanto-Shinetsu) |  |  |  |  |  |  |  |  |  |
| Hokkaido |  |  |  |  |  |  |  |  |  |
| Tohoku |  |  |  |  |  |  |  |  |  |
| Tokai-Hokuriku |  |  |  |  |  |  |  |  |  |
| Kinki |  |  |  |  |  |  |  |  |  |
| Chugoku-Shikoku |  |  |  |  |  |  |  |  |  |
| Kyushu |  |  |  |  |  |  |  |  |  |
| **Random effects** | **Model 1 (Empty model)** | | | **Model 2 (Municipality)** | | | **Model 3 (SMA)** | | |
| **Municipality-level** |  |  |  |  |  |  |  |  |  |
| Variance (SE) | 3398 | (121) |  | 3222 | (115) |  | 3381 | (120) |  |
| VPC (Variance Partition Coefficient) | 0.29 |  |  | 0.30 |  |  | 0.43 |  |  |
| Explained variance: %  (i.e., Proportional Change in Variance) | Ref. |  |  | 5.2 |  |  | 0.5 |  |  |
| **Secondary medical area-level** |  |  |  |  |  |  |  |  |  |
| Variance (SE) | 1863 | (212) |  | 1615 | (191) |  | 1093 | (145) |  |
| VPC | 0.16 |  |  | 0.15 |  |  | 0.14 |  |  |
| Explained variance: % | Ref. |  |  | 13.3 |  |  | 41.3 |  |  |
| **Prefecture-level variance** |  |  |  |  |  |  |  |  |  |
| Variance (SE) | 6478 | (1417) |  | 5896 | (1299) |  | 3355 | (776) |  |
| VPC | 0.55 |  |  | 0.55 |  |  | 0.43 |  |  |
| Explained variance: % | Ref. |  |  | 9.0 |  |  | 48.2 |  |  |
| **Total** |  |  |  |  |  |  |  |  |  |
| Variance (sum of three levels) | 11739 |  |  | 10733 |  |  | 7829 |  |  |
| Explained variance: % | Ref. |  |  | 8.6 |  |  | 33.3 |  |  |
| **Model fit statistics** |  |  |  |  |  |  |  |  |  |
| Log-likelihood | -10643 |  |  | -10582 |  |  | -10573 |  |  |
| AIC | 21293 |  |  | 21192 |  |  | 21168 |  |  |
| BIC | 21315 |  |  | 21270 |  |  | 21229 |  |  |

**Supplementary Table 4. continued.**

|  | **Model 4** | | | **Model 5** | | | **Model 6** | | |
| --- | --- | --- | --- | --- | --- | --- | --- | --- | --- |
|  | **Prefecture-level variable** | | | **Secondary medical-area and prefecture-level variables** | | | **Full model with all variables** | | |
| **Fixed effects** | **B** | **95% CI** | **p-value** | **B** | **95% CI** | **p-value** | **B** | **95% CI** | **p-value** |
| **Municipality-level variables** |  |  |  |  |  |  |  |  |  |
| Proportion of population aged 75 years or above |  |  |  |  |  |  | -1.68 | (-2.60, -0.76) | <0.001 |
| Proportion of workers in primary industry |  |  |  |  |  |  | -0.40 | (-0.85, 0.06) | 0.09 |
| Unemployment rate |  |  |  |  |  |  | -0.26 | (-3.28, 2.76) | 0.87 |
| Population density |  |  |  |  |  |  | -0.01 | (-0.03, 0.002) | 0.09 |
| Taxable income per tax payer |  |  |  |  |  |  | -0.22 | (-1.18, 0.75) | 0.66 |
| Male life expectancy |  |  |  |  |  |  | -8.41 | (-15.15, -1.67) | 0.01 |
| Female life expectancy |  |  |  |  |  |  | 10.60 | (2.16, 19.03) | 0.01 |
| Proportion of older adults certified as needing long-term care |  |  |  |  |  |  | 5.65 | (3.82, 7.48) | <0.001 |
| Long-term care benefit expenditure |  |  |  |  |  |  | -0.06 | (-0.08, -0.04) | <0.001 |
| Outpatient medical care expenditure |  |  |  |  |  |  | 0.16 | (0.08, 0.24) | <0.001 |
| **Secondary medical area-level variables** |  |  |  |  |  |  |  |  |  |
| Number of hospital beds for general care |  |  |  | 0.03 | (0.002, 0.06) | 0.04 | 0.03 | (0.002, 0.06) | 0.04 |
| Number of hospital beds for psychiatric care |  |  |  | 0.05 | (0.02, 0.09) | 0.002 | 0.05 | (0.01, 0.08) | 0.005 |
| Number of hospital beds for chronic care |  |  |  | 0.08 | (0.04, 0.12) | <0.001 | 0.09 | (0.05, 0.13) | <0.001 |
| Average number of days in hospital |  |  |  | 0.75 | (-0.02, 1.52) | 0.06 | 0.77 | (0.04, 1.50) | 0.04 |
| Number of doctors |  |  |  | 0.09 | (0.03, 0.16) | 0.007 | 0.08 | (0.01, 0.14) | 0.03 |
| Number of home visiting care by doctor |  |  |  | 0.02 | (0.01, 0.04) | 0.009 | 0.01 | (-0.01, 0.02) | 0.22 |
| Number of end-of-life care at home |  |  |  | -2.10 | (-3.43, -0.77) | 0.002 | -1.66 | (-2.94, -0.38) | 0.01 |
| **Prefecture-level variable (RHWB regions)** |  |  |  |  |  |  |  |  |  |
| (Reference: Kanto-Shinetsu) |  |  |  |  |  |  |  |  |  |
| Hokkaido | 158.46 | (88.20, 228.71) | <0.001 | 105.02 | (44.51, 165.53) | 0.001 | 91.75 | (31.33, 152.18) | 0.003 |
| Tohoku | -34.75 | (-73.00, 3.50) | 0.08 | -38.03 | (-70.62, -5.44) | 0.02 | -45.18 | (-77.83, -12.52) | 0.007 |
| Tokai-Hokuriku | 27.00 | (-11.63, 65.62) | 0.17 | 17.78 | (-14.77, 50.33) | 0.28 | 17.22 | (-14.95, 49.39) | 0.29 |
| Kinki | 87.19 | (50.78, 123.60) | <0.001 | 77.04 | (46.29, 107.8) | <0.001 | 62.38 | (31.94, 92.82) | <0.001 |
| Chugoku-Shikoku | 122.74 | (88.00, 157.48) | <0.001 | 69.86 | (38.93, 100.79) | <0.001 | 59.59 | (28.67, 90.52) | <0.001 |
| Kyushu | 178.43 | (143.68, 213.18) | <0.001 | 121.65 | (90.77, 152.53) | <0.001 | 118.21 | (87.4, 149.03) | <0.001 |
| **Random effects** | **Model 4 (Prefecture)** | | | **Model 5 (SMA & Prefecture)** | | | **Model 6 (Full model)** | | |
| **Municipality-level** |  |  |  |  |  |  |  |  |  |
| Variance (SE) | 3400 | (121) |  | 3382 | (120) |  | 3209 | (114) |  |
| VPC (Variance Partition Coefficient) | 0.54 |  |  | 0.65 |  |  | 0.66 |  |  |
| Explained variance: %  (i.e., Proportional Change in Variance) | -0.1 |  |  | 0.5 |  |  | 5.6 |  |  |
| **Secondary medical area-level** |  |  |  |  |  |  |  |  |  |
| Variance (SE) | 1868 | (213) |  | 1091 | (145) |  | 924 | (130) |  |
| VPC | 0.30 |  |  | 0.21 |  |  | 0.19 |  |  |
| Explained variance: % | -0.3 |  |  | 41.4 |  |  | 50.4 |  |  |
| **Prefecture-level variance** |  |  |  |  |  |  |  |  |  |
| Variance (SE) | 1039 | (309) |  | 738 | (220) |  | 731 | (214) |  |
| VPC | 0.16 |  |  | 0.14 |  |  | 0.15 |  |  |
| Explained variance: % | 84.0 |  |  | 88.6 |  |  | 88.7 |  |  |
| **Total** |  |  |  |  |  |  |  |  |  |
| Variance (sum of three levels) | 6307 |  |  | 5211 |  |  | 4864 |  |  |
| Explained variance: % | 46.3 |  |  | 55.6 |  |  | 58.6 |  |  |
| **Model fit statistics** |  |  |  |  |  |  |  |  |  |
| Log-likelihood | -10607 |  |  | -10543 |  |  | -10484 |  |  |
| AIC | 21234 |  |  | 21120 |  |  | 21023 |  |  |
| BIC | 21289 |  |  | 21215 |  |  | 21173 |  |  |

B, unstandardized beta coefficient; CI, confidence interval

**Supplementary Table 5. Multilevel Linear Regression Models of Regional Disparity Index of Inpatient Medical Expenditure (FY 2018) for Adults Aged 75 Years and Above in Association with Municipality-, Secondary Medical Area-, and Prefecture-level Variables.**

|  | **Model 1** | | | **Model 2** | | | **Model 3** | | |
| --- | --- | --- | --- | --- | --- | --- | --- | --- | --- |
|  | **Empty model** | | | **Municipality-level variables** | | | **Secondary medical area-level variables** | | |
| **Fixed effects** | **B** | **95% CI** | **p-value** | **B** | **95% CI** | **p-value** | **B** | **95% CI** | **p-value** |
| **Municipality-level variables** |  |  |  |  |  |  |  |  |  |
| Proportion of population aged 75 years or above |  |  |  | -0.28 | (-0.47, -0.09) | 0.004 |  |  |  |
| Proportion of workers in primary industry |  |  |  | -0.15 | (-0.24, -0.05) | 0.002 |  |  |  |
| Unemployment rate |  |  |  | 0.13 | (-0.50, 0.75) | 0.69 |  |  |  |
| Population density |  |  |  | -0.004 | (-0.01, -0.0001) | 0.047 |  |  |  |
| Taxable income per tax payer |  |  |  | -0.06 | (-0.25, 0.14) | 0.57 |  |  |  |
| Male life expectancy |  |  |  | -1.55 | (-2.94, -0.15) | 0.03 |  |  |  |
| Female life expectancy |  |  |  | 1.90 | (0.16, 3.64) | 0.03 |  |  |  |
| Proportion of older adults certified as needing long-term care |  |  |  | 0.76 | (0.35, 1.16) | ＜0.001 |  |  |  |
| Long-term care benefit expenditure |  |  |  | -0.02 | (-0.02, -0.01) | ＜0.001 |  |  |  |
| Outpatient medical care expenditure |  |  |  | 0.13 | (0.06, 0.20) | ＜0.001 |  |  |  |
| **Secondary medical area-level variables** |  |  |  |  |  |  |  |  |  |
| Number of hospital beds for general care |  |  |  |  |  |  | 0.004 | (-0.002, 0.01) | 0.21 |
| Number of hospital beds for psychiatric care |  |  |  |  |  |  | 0.01 | (0.005, 0.02) | 0.001 |
| Number of hospital beds for chronic care |  |  |  |  |  |  | 0.01 | (0.003, 0.02) | 0.008 |
| Average number of days in hospital |  |  |  |  |  |  | 0.20 | (0.04, 0.37) | 0.01 |
| Number of doctors |  |  |  |  |  |  | 0.03 | (0.01, 0.04) | ＜0.001 |
| Number of home visiting care by doctor |  |  |  |  |  |  | 0.005 | (0.002, 0.01) | 0.004 |
| Number of end-of-life care at home |  |  |  |  |  |  | -0.56 | (-0.84, -0.28) | ＜0.001 |
| **Prefecture-level variable (RHWB regions)** |  |  |  |  |  |  |  |  |  |
| (Reference: Kanto-Shinetsu) |  |  |  |  |  |  |  |  |  |
| Hokkaido |  |  |  |  |  |  |  |  |  |
| Tohoku |  |  |  |  |  |  |  |  |  |
| Tokai-Hokuriku |  |  |  |  |  |  |  |  |  |
| Kinki |  |  |  |  |  |  |  |  |  |
| Chugoku-Shikoku |  |  |  |  |  |  |  |  |  |
| Kyushu |  |  |  |  |  |  |  |  |  |
| **Random effects** | **Model 1 (Empty model)** | | | **Model 2 (Municipality)** | | | **Model 3 (SMA)** | | |
| **Municipality-level** |  |  |  |  |  |  |  |  |  |
| Variance (SE) | 138 | (5) |  | 129 | (5) |  | 138 | (5) |  |
| VPC (Variance Partition Coefficient) | 0.28 |  |  | 0.29 |  |  | 0.41 |  |  |
| Explained variance: %  (i.e., Proportional Change in Variance) | Ref. |  |  | 6.5 |  |  | 0.0 |  |  |
| **Secondary medical area-level** |  |  |  |  |  |  |  |  |  |
| Variance (SE) | 81 | (9) |  | 66 | (8) |  | 48 | (6) |  |
| VPC | 0.17 |  |  | 0.15 |  |  | 0.14 |  |  |
| Explained variance: % | Ref. |  |  | 18.5 |  |  | 40.7 |  |  |
| **Prefecture-level variance** |  |  |  |  |  |  |  |  |  |
| Variance (SE) | 268 | (59) |  | 252 | (55) |  | 149 | (34) |  |
| VPC | 0.55 |  |  | 0.56 |  |  | 0.44 |  |  |
| Explained variance: % | Ref. |  |  | 6.0 |  |  | 44.4 |  |  |
| **Total** |  |  |  |  |  |  |  |  |  |
| Variance (sum of three levels) | 487 |  |  | 447 |  |  | 335 |  |  |
| Explained variance: % | Ref. |  |  | 8.2 |  |  | 31.2 |  |  |
| **Model fit statistics** |  |  |  |  |  |  |  |  |  |
| Log-likelihood | -7628 |  |  | -7550 |  |  | -7561 |  |  |
| AIC | 15263 |  |  | 15128 |  |  | 15145 |  |  |
| BIC | 15286 |  |  | 15205 |  |  | 15206 |  |  |

**Supplementary Table 5. continued.**

|  | **Model 4** | | | **Model 5** | | | **Model 6** | | |
| --- | --- | --- | --- | --- | --- | --- | --- | --- | --- |
|  | **Prefecture-level variable** | | | **Secondary medical-area and prefecture-level variables** | | | **Full model with all variables** | | |
| **Fixed effects** | **B** | **95% CI** | **p-value** | **B** | **95% CI** | **p-value** | **B** | **95% CI** | **p-value** |
| **Municipality-level variables** |  |  |  |  |  |  |  |  |  |
| Proportion of population aged 75 years or above |  |  |  |  |  |  | -0.35 | (-0.54, -0.17) | ＜0.001 |
| Proportion of workers in primary industry |  |  |  |  |  |  | -0.16 | (-0.25, -0.07) | 0.001 |
| Unemployment rate |  |  |  |  |  |  | -0.17 | (-0.78, 0.44) | 0.58 |
| Population density |  |  |  |  |  |  | -0.002 | (-0.01, 0.001) | 0.24 |
| Taxable income per tax payer |  |  |  |  |  |  | -0.08 | (-0.28, 0.11) | 0.40 |
| Male life expectancy |  |  |  |  |  |  | -1.61 | (-2.96, -0.25) | 0.02 |
| Female life expectancy |  |  |  |  |  |  | 1.88 | (0.18, 3.57) | 0.03 |
| Proportion of older adults certified as needing long-term care |  |  |  |  |  |  | 0.73 | (0.34, 1.12) | ＜0.001 |
| Long-term care benefit expenditure |  |  |  |  |  |  | -0.02 | (-0.02, -0.01) | ＜0.001 |
| Outpatient medical care expenditure |  |  |  |  |  |  | 0.11 | (0.04, 0.18) | 0.001 |
| **Secondary medical area-level variables** |  |  |  |  |  |  |  |  |  |
| Number of hospital beds for general care |  |  |  | 0.003 | (-0.003, 0.01) | 0.29 | 0.004 | (-0.001, 0.01) | 0.13 |
| Number of hospital beds for psychiatric care |  |  |  | 0.01 | (0.005, 0.02) | 0.001 | 0.01 | (0.03, 0.02) | 0.003 |
| Number of hospital beds for chronic care |  |  |  | 0.01 | (0.004, 0.02) | 0.004 | 0.01 | (0.01, 0.02) | 0.001 |
| Average number of days in hospital |  |  |  | 0.16 | (0.01, 0.32) | 0.05 | 0.18 | (0.03, 0.33) | 0.02 |
| Number of doctors |  |  |  | 0.03 | (0.01, 0.04) | ＜0.001 | 0.02 | (0.01, 0.04) | 0.001 |
| Number of home visiting care by doctor |  |  |  | 0.004 | (0.001, 0.01) | 0.007 | 0.002 | (-0.001, 0.005) | 0.21 |
| Number of end-of-life care at home |  |  |  | -0.54 | (-0.82, -0.27) | ＜0.001 | -0.41 | (-0.66, -0.15) | 0.002 |
| **Prefecture-level variable (RHWB regions)** |  |  |  |  |  |  |  |  |  |
| (Reference: Kanto-Shinetsu) |  |  |  |  |  |  |  |  |  |
| Hokkaido | 28.58 | (14.00, 43.15) | <0.001 | 18.9 | (6.26, 31.53) | 0.003 | 16.82 | (4.12, 29.51) | 0.009 |
| Tohoku | -9.49 | (-17.42, -1.55) | 0.02 | -9.82 | (-16.62, -3.03) | 0.005 | -9.52 | (-16.33, -2.71) | 0.006 |
| Tokai-Hokuriku | 5.45 | (-2.56, 13.46) | 0.18 | 3.87 | (-2.92, 10.66) | 0.26 | 3.73 | (-2.99, 10.44) | 0.28 |
| Kinki | 18.87 | (11.33, 26.42) | <0.001 | 17.09 | (10.67, 23.5) | <0.001 | 13.83 | (7.46, 20.20) | <0.001 |
| Chugoku-Shikoku | 23.00 | (15.80, 30.20) | <0.001 | 13.03 | (6.58, 19.47) | <0.001 | 12.30 | (5.88, 18.72) | <0.001 |
| Kyushu | 35.30 | (28.10, 42.51) | <0.001 | 24.35 | (17.92, 30.78) | <0.001 | 24.97 | (18.55, 31.39) | <0.001 |
| **Random effects** | **Model 4 (Prefecture)** | | | **Model 5 (SMA & Prefecture)** | | | **Model 6 (Full model)** | | |
| **Municipality-level** |  |  |  |  |  |  |  |  |  |
| Variance (SE) | 138 | (5) |  | 138 | (5) |  | 129 | (5) |  |
| VPC (Variance Partition Coefficient) | 0.52 |  |  | 0.63 |  |  | 0.65 |  |  |
| Explained variance: %  (i.e., Proportional Change in Variance) | 0.0 |  |  | 0.0 |  |  | 6.5 |  |  |
| **Secondary medical area-level** |  |  |  |  |  |  |  |  |  |
| Variance (SE) | 82 | (9) |  | 48 | (6) |  | 37 | (5) |  |
| VPC | 0.31 |  |  | 0.22 |  |  | 0.19 |  |  |
| Explained variance: % | -1.2 |  |  | 40.7 |  |  | 54.3 |  |  |
| **Prefecture-level variance** |  |  |  |  |  |  |  |  |  |
| Variance (SE) | 45 | (13) |  | 32 | (10) |  | 33 | (9) |  |
| VPC | 0.17 |  |  | 0.15 |  |  | 0.17 |  |  |
| Explained variance: % | 83.2 |  |  | 88.1 |  |  | 87.7 |  |  |
| **Total** |  |  |  |  |  |  |  |  |  |
| Variance (sum of three levels) | 265 |  |  | 218 |  |  | 199 |  |  |
| Explained variance: % | 45.6 |  |  | 55.2 |  |  | 59.1 |  |  |
| **Model fit statistics** |  |  |  |  |  |  |  |  |  |
| Log-likelihood | -7593 |  |  | -7531 |  |  | -7452 |  |  |
| AIC | 15206 |  |  | 15097 |  |  | 14958 |  |  |
| BIC | 15261 |  |  | 15191 |  |  | 15107 |  |  |

B, unstandardized beta coefficient; CI, confidence interval

**Supplementary References**

1. Ministry of Internal Affairs and Communications, Japan. Basic Resident Register: Population and Households. (2018). https://www.soumu.go.jp/main_sosiki/jichi_gyousei/daityo/jinkou_jinkoudoutai-setaisuu.html [Accessed February 2, 2023].
2. Cabinet Office, Japan. Prefectural Economic Accounts. (2018). <https://www.esri.cao.go.jp/jp/sna/data/data_list/kenmin/files/contents/main_2020.html> [Accessed November 21, 2023]
3. Ministry of Health, Labour and Welfare, Japan. Analysis of Regional Differences in Medical Care Expenditures. (2017, 2018). https://www.mhlw.go.jp/stf/seisakunitsuite/bunya/kenkou_iryou/iryouhoken/database/iryomap/index.html [Accessed January 6, 2023].
4. Statistics Bureau, Ministry of Internal Affairs and Communications, Japan. 2015 Population Census. (2015). https://www.stat.go.jp/english/data/kokusei/2015/summary.html [Accessed January 18, 2023].
5. Statistics Bureau, Ministry of Internal Affairs and Communications, Japan. Municipality Taxation Status. (2015). https://www.e-stat.go.jp/regional-statistics/ssdsview/municipality [Accessed January 10, 2023].
6. Ministry of Health, Labour and Welfare, Japan. Overview of Municipality Life Tables. (2015). https://www.mhlw.go.jp/toukei/saikin/hw/life/ckts15/index.html [Accessed January 18, 2023].
7. Ministry of Health, Labour and Welfare, Japan. Long-Term Care Insurance Business Status Report. (2017, 2018). https://www.mhlw.go.jp/topics/kaigo/toukei/joukyou.html [Accessed January 27, 2023].
8. Ministry of Health, Labour and Welfare, Japan. Statistics of Physicians, Dentists, and Pharmacists. (2016). https://www.mhlw.go.jp/toukei/saikin/hw/ishi/16/index.html [Accessed January 24, 2023].
9. Ministry of Health, Labour and Welfare, Japan. Hospital Report. (2017). https://www.mhlw.go.jp/toukei/list/80-1.html [Accessed January 26, 2023].
10. Ministry of Health, Labour and Welfare, Japan. Survey of Medical Institutions. (2017). https://www.mhlw.go.jp/toukei/list/79-1.html [Accessed February 7, 2023].
